# Stepwise accumulation of mutations in CesA3 in *Phytophthora sojae* results in increasing resistance to CAA fungicides

**DOI:** 10.1111/eva.13176

**Published:** 2020-12-31

**Authors:** Meng Cai, Can Zhang, Weizhen Wang, Qin Peng, Xi Song, Brett M. Tyler, Xili Liu

**Affiliations:** ^1^ College of Plant Protection China Agricultural University Beijing China; ^2^ College of Chemistry Key Laboratory of Pesticide & Chemical Biology of Ministry of Education Central China Normal University Wuhan China; ^3^ Department of Botany & Plant Pathology Oregon State University Corvallis Oregon USA

**Keywords:** CAA fungicides, CesA3, flumorph, *Phytophthora sojae*, resistance, stepwise accumulation of mutations

## Abstract

Flumorph is a carboxylic acid amide (CAA) fungicide with high activity against oomycetes. However, evolution to CAAs from low resistance to high resistance has never been reported. This study investigated the basis of resistance evolution of flumorph in *Phytophthora sojae*. Total of 120 *P. sojae* isolates were collected and their sensitivity to flumorph was evaluated. Although no spontaneous resistance was found among the field isolates, adaptation on flumorph‐amended media resulted in the selection of five stable mutant types exhibiting varying degrees of resistance to CAAs. Type I, which exhibited the lowest resistance level, was obtained when the wild‐type isolate was exposed to a low concentration of flumorph, but no resistant mutants were obtained by direct exposure to higher concentrations. However, the more resistant types (Type II, III, IV and V) were obtained when Type I were exposed to higher concentrations of flumorph. Similar results were obtained when the entire screening process was repeated, which implied that evolution of resistance to flumorph in *P. sojae* could be a two‐step process, where high resistance phenotypes could develop gradually from low resistance ones. Further investigation into molecular mechanism strongly confirmed that evolution of isolates highly resistant to flumorph occurs in a stepwise process with Type I as intermediary, through accumulation of mutations in their target protein of CAAs (CesA3). Together, our findings indicate that application of low rates of flumorph in field could result in selection of low resistance Type I isolates, but that raising dosage to maintain comparable levels of control could elicit rapid evolution of more resistant Type II, III, IV and V isolates with stepwise accumulation of mutations in CesA3, which would render flumorph ineffective as a control method. Precautionary resistance management strategy should be implemented. The phenomenon described in the study could have broader biological significance.

## 1 BACKGROUND

Oomycetes are destructive pathogens in agriculture, aquaculture and forestry, as well as to natural ecosystems. For example, the oomycete genus *Phytophthora* contains more than 100 plant pathogenic species that infect a huge range of economically important plant species such as oak, potato, soybean, pepper, tomato and pineapple (Kamoun et al., 2015). The soybean root rot disease caused by *Phytophthora sojae* leads to annual losses of $200–300 million in the US alone, with global losses estimated at $1–2 billion per year (Tyler, 2007).

Application of anti‐oomycete chemicals is one of the most effective methods to control oomycete diseases (Whisson et al., 2011). However, oomycetes are phylogenetically distinct from true fungi due to many unique biochemical characteristics in their life cycle, such as their diploid property, having cellulose but no chitin as a cell wall component, having almost no sterol in the cell membrane, and producing lysine along the *α*‐*ε*‐diaminopimelic acid pathway (Beakes et al., 2012). Thus, many conventional fungicides that are effective against true fungi are ineffective against oomycete diseases, such as sterol demethylation inhibitor fungicides, methyl benzimidazole carbamate fungicides and succinate dehydrogenase inhibitor fungicides (Gisi & Sierotzki, 2015). The carboxylic acid amide (CAA) fungicides, comprising the three subclasses of cinnamic acid amides (dimethomorph, flumorph and pyrimorph), valinamide carbamates (iprovalicarb and benthiavalicarb), and mandelic acid amides (mandipropamid), have shown specific activity against most oomycete pathogens, such as *Phytophthora* species, *Plasmopara viticola*, *Bremia lactucae* and *Pseudoperonospora cubensis* (Gisi et al., 2012). Despite differences in the chemical structures of the three subclasses, cross‐resistance exists among all group members in the vast majority of the tested oomycete isolates (Gisi et al., 2007). Thus, CAA resistance could be a severe problem once resistance to one of the CAA members arises. Several studies have revealed that resistance to CAAs has already developed in field populations of oomycete pathogens including *P. viticola* (Sierotzki et al., 2011) and *P. cubensis* (Zhu et al., 2007). The resistance risk for CAA fungicides in *P. viticola* and *P. cubensis* was classified by the Fungicide Resistance Action Committee (FRAC) as moderate due to the degree of resistance observed and the reproductive strategies of these pathogens (www.frac.info). Surprisingly, despite a history of use spanning 25 years, no resistant isolates have yet been detected in field populations of *P. infestans*, according to the report of FRAC CAA working group in 2018 (www.frac.info). Furthermore, although some resistant *Phytophthora* mutants have been produced under laboratory conditions, the frequency of resistance was extremely low and often incurred a fitness penalty (Chen et al., 2012; Cohen et al., 2007; Pang et al., 2013; Rubin et al., 2008). Consequently, the resistance risk for CAA fungicides in *P. infestans* (Cohen et al., 2007; Rubin et al., 2008), *P. capsici* (Lu et al., 2010) and *P. melonis* (Chen et al., 2012; Pang et al., 2013) has been classified as low to medium. Different oomycete species can vary in their CAA resistance risk and their resistance evolution process. To date, there have been no reports about resistance evolution of CAA fungicides in any oomycete pathogens.

Carboxylic acid amide fungicides are potent disrupters of cellulose biosynthesis, which act by binding to the cellulose synthase 3 enzyme (CesA3; Blum et al., 2012). Molecular analyses of resistant mechanisms in oomycete pathogens have revealed that single amino acid substitutions in the CesA3 protein can confer resistance to CAAs. For example, in resistant field isolates of *P. viticola* (Blum, Waldner, et al., 2010; Sawant et al., 2017; Sierotzki et al., 2011) and *P. cubensis* (Blum et al., 2011; Sierotzki et al., 2011), the replacement of a conserved glycine at position 1105 by either serine, valine or tryptophan (G1105S/V/W) resulted in resistance to all the CAA fungicides tested. Similarly, laboratory mutants of *P. infestans* were found to contain G1105A/V mutations in CesA3 (Blum, Boehler, et al., 2010). In artificial mutants, two amino acid substitutions at position 1109, V1109L and V1109M, were also identified to be responsible for CAA resistance (Blum & Gisi, 2012; Chen et al., 2011, 2012), while another amino acid replacement, Q1077K, was reported to be responsible for CAA resistance in *P. capsici* (Pang et al., 2013). However, although the mutations listed above have all been putatively linked to CAA resistance, to date there has been no direct verification of their role or interrelations in CAA resistance, or a precise description of the resistance mechanism. The objectives of the current study were therefore to investigate the CAA resistance development in *P. sojae*, specifically to (1) examine the resistance evolution process for the CAA fungicide flumorph by exposure of *P. sojae* wild‐type isolates to a set of different concentrations of flumorph, (2) investigate the molecular basis for flumorph resistance and (3) validate the role of any mutations discovered.

## 2 MATERIALS AND METHODS

### 2.1 Isolates and culture conditions

The wild‐type *P. sojae* isolates used in this study are listed in Table S1. All the isolates were routinely dark‐cultured on a V8 medium (200 ml/L V8 original 100% vegetable juice from Campbell Soup Company, 1 g/L CaCO_3_, and 15 g/L agar) at 25°C and maintained in 5‐ml plastic tubes containing V8 medium slants kept under mineral oil at 18°C for long‐term storage.

### 2.2 Fungicide sensitivity assays

Analytical‐grade samples of the fungicides flumorph, dimethomorph, mandipropamid and iprovalicarb were purchased from Sigma‐Aldrich and dissolved in dimethyl sulfoxide, with the final concentration of the solvent limited to 0.1% (v/v). The sensitivity tests were performed using mycelial growth assays. Mycelia plugs (5 mm in diameter) were excised from the growing edges of 5‐day‐old colonies of *P. sojae* and transferred to the centre of fresh V8 plates amended with individual fungicides at the range of concentrations listed in Table S2. Three replicate plates were used for each treatment. The effect of each fungicide on the mycelial growth of *P. sojae* was determined by measuring the colony diameters after 4 days of dark incubation at 25°C. The data collected were then subjected to linear regression of the probit of growth inhibition ratio against the logarithmic value (base 10) of fungicide concentration in excel according to the method of a previous study (Chen et al., 2012), and the effective concentration for 50% inhibition (EC_50_) calculated from the resulting dose–response curves after probit analysis.

### 2.3 Generation of flumorph‐resistant *P. sojae* mutants

Six wild‐type *P. sojae* isolates (Ps4, Ps5, Ps6, Ps8, Ps10 and Ps13) were randomly selected from the strains in Table S1 for screening experiments to select flumorph‐resistant mutants. Per wild‐type isolate, 15 replicates of 9‐cm mycelia plates were used. Mycelial plugs were cut from 5‐day‐old colonies and transferred to fresh V8 plates containing 2.5, 5 or 100 μg/ml of flumorph. In the first round of adaption, per wild‐type isolate per concentration, about 500 mycelial plugs (five replicate 15‐cm plates) were inoculated in total. The plates were then dark‐incubated at 25°C for 15–30 days, before the area containing the fastest‐growing portion of the colony was transferred to fresh V8 plates amended with the same concentration of flumorph to stabilize any resistance. The newly grown colonies were then subjected to further rounds of subculture amended with increasing concentrations of flumorph (5 or 100 μg/ml) to induce mutants with higher levels of resistance (Figure S1). If no fastest‐growing portion was identified, the whole plate of mycelia was cut into as many mycelial plugs as possible and then transferred to new plates amended with the same or increasing concentrations of flumorph (5 or 100 μg/ml; Figure S1). In total, about 10 rounds of subculture were transferred. The resistance factor (RF) for each mutant was calculated by dividing the EC_50_ of the corresponding wild‐type parental isolate by the EC_50_ of the mutant. The screening process was repeated.

### 2.4 Biological characteristics of flumorph‐resistant mutants

#### 2.4.1 Stability of resistance in mutant isolates

The flumorph‐resistant mutants and their corresponding parent isolates were subjected to 10 successive transfers on fungicide‐free V8 plates, by excising mycelial plugs from the edges of 5‐day‐old colonies and transferring them to fresh plates (one plug per plate), which were dark‐incubated at 25°C. Three replicate plates were prepared for each isolate, and the entire experiment was conducted twice. The EC_50_ values of each isolate were determined after the first, third, fifth, seventh and tenth subcultures.

#### 2.4.2 Mycelial growth rate

The mycelial growth rates of the mutants and their parental isolates were compared by dark incubation on V8 plates at their optimum growth temperature of 25°C. The colony diameter was measured in two perpendicular directions after 5 days of incubation. Each isolate or mutant was represented by three replicate plates, and the entire experiment was performed twice.

#### 2.4.3 Sporulation and cyst germination

The zoospore production of the flumorph‐resistant mutants and their parental isolates were compared after 5 days of dark incubation on V8 media at 25°C. The production of zoospores was induced by repeatedly washing the plates with distilled water as described in a previous study (Morris & Ward, 1992). Each mutant or parental isolate was represented by 10 replicate plates, and the entire was experiment conducted twice. Sporulation was quantified using a hemocytometer and expressed as the number of zoospores per square centimetre of culture. The germination of the cystospores was then determined by microscopy after 12 h of dark incubation on V8 agar at 25°C, following the protocol described in another study (Bi et al., 2011).

#### 2.4.4 Virulence assay

The virulence of the mutants and their parental isolates were compared by inoculating soybean seedlings (cv. Williams) with 10 μl of a zoospore suspension containing 10^5^ zoospores/ml (Dou et al., 2008). Newly grown seedlings (ca. 7–10 days old) were used for the assays, and the lesion length was measured after 2–3 days of dark incubation at 25°C. Ten seedlings were used for each isolate or mutant, and the entire experiment was performed twice.

### 2.5 Cross‐resistance assay

Mycelial growth assays were used to determine whether the flumorph‐resistant mutants exhibited cross‐resistance to fungicides with different modes of action and belonging to different chemical groups. All of the compounds selected (chlorothalonil, azoxystrobin, cymoxanil, metalaxyl and zoxamide) are currently marketed for the control of oomycete plant pathogens. The EC_50_ values for each mutant–fungicide combination were determined using the mycelia growth inhibition assay described above, with the concentrations used for each fungicide listed in Table S2.

### 2.6 Nucleic acid manipulations

The total DNA samples were extracted from the mycelia of *P. sojae* according to the protocol of a previous study (Chen et al., 2012), while total RNA was extracted from lyophilized mycelial tissue using the SV Total RNA Isolation System according to the protocol of the manufacturer (Promega Biotechnology Co., Ltd.), and first‐strand cDNA synthesized using the PrimeScript™ RT Reagent Kit with gDNA Eraser (Perfect Real Time; Takara Biotechnology Co., Ltd.). The PCR reactions were performed in a Bio‐Rad MyCycler™ Thermocycler, using Takara PrimeSTAR HS DNA Polymerase (Takara Biotechnology Co., Ltd.) according to the protocol of the manufacturer in conjunction with the primers listed in Table S3. The PCR products were purified using the EasyPure^®^ PCR Purification Kit (TransGen Biotech Co.) and sequenced by Beijing Sunbiotech Co., Ltd. The resulting sequence data were analysed using DNAMAN (version 8) and Chromas (version 2.23) software.

### 2.7 Transformation of *P. sojae*

The sgRNA target sites were selected using the *EuPaGDT* tool (http://grna.ctegd.uga.edu/) developed by Peng (Peng & Tarleton, 2015), with the off‐target analysis performed simultaneously. The secondary structures of the sgRNA sequences were analysed using the *RNA structure* tool hosted on the University of Rochester Medical Center website (http://rna.urmc.rochester.edu/RNAstructureWeb/Servers/Predict1/Predict1.html). The sgRNA sequences were then cloned into the *Nhe*I (NEB, R3131S) and *Bsa*I (NEB, R3535S) sites of the pYF2.3G‐Ribo‐sgRNA plasmid according to the protocol of a previous study (Fang & Tyler, 2016). The primers used for the construction of the sgRNA vectors are listed in Table S3.

The transformation vector for the homology directed repair was prepared by cloning a 1305‐bp fragment of the *CesA3* gene from the wild‐type *P. sojae* isolate P6497 into the *Eco*RV (NEB, R3195S) site of the pBluescript II KS + vector, using the In‐Fusion^®^ HD Cloning Kit (Clontech). The primers PsCesA3.HDT.F1 and PsCesA3.HDT.R1 used for the amplification of the 1305‐bp homologous donor template are listed in Table S3. Mutations were then introduced using the QuikChange Lightning Multi Site‐Directed Mutagenesis Kit (Agilent Technologies). Five individual mutations were evaluated (I1027V, V1025L, G1020A, G1020S and Q992H), as well as four more comprising either V1025L, G1020A, G1020S or Q992H in combination with I1027V. The six primers used to mediate the mutations are listed in Table S3. All enzyme digestions and ligations were performed according to the protocol of the manufacturer (New England BioLabs^®^ Inc), and the integrity and accuracy of the inserts confirmed by DNA sequencing.

The transformation was accomplished using the improved polyethylene glycol‐mediated protoplast protocol detailed in a previous study (Fang & Tyler, 2016). The transformants were initially screened in a pea broth medium amended with 30 μg/ml geneticin (AG Scientific) and then on V8 agar plates containing 50 μg/ml geneticin. The CAA sensitivity of the resulting transformants was then assessed using the mycelial growth inhibition assay described above.

### 2.8 Data analysis

Statistical analyses were performed using SPSS Statistics software (version 20; IBM Corp.). One‐way ANOVA was used with a Tukey's honestly significant difference test (Tukey's HSD; *p* = 0.05) to compare the mean values of the tested biological parameters among different resistance isolates.

## 3 RESULTS

### 3.1 Sensitivity of *P. sojae* field population to flumorph

**Figure 1:**
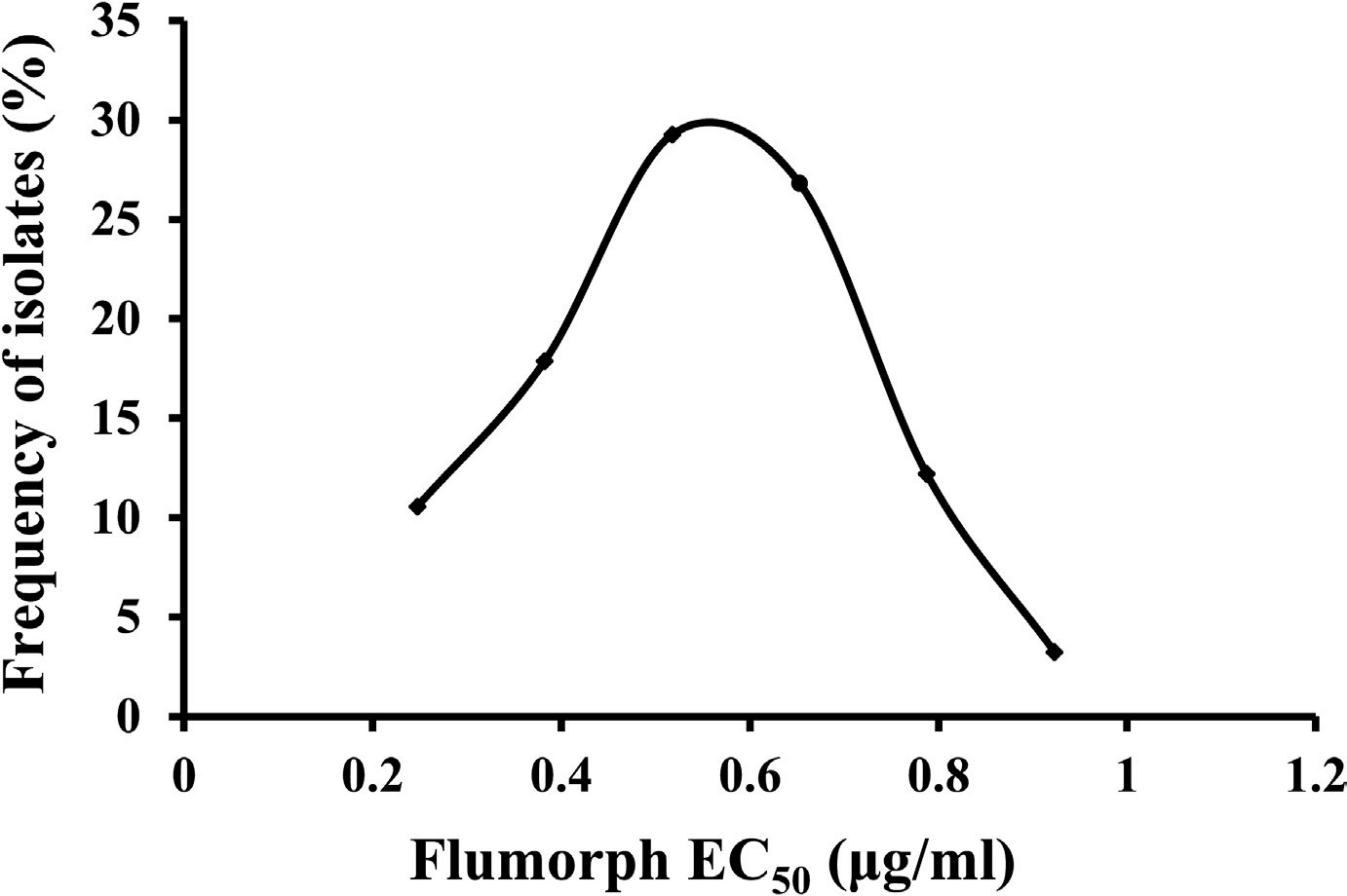
Frequency distribution of flumorph EC_50_ values for 120 field isolates of *Phytophthora sojae*

The flumorph sensitivity of the 120 *P. sojae* field isolates tested varied, with EC_50_ values ranging from 0.18 to 0.99 μg/ml and a mean of 0.54 (±0.01) μg/ml (Table S1). This equates to a 5.5‐fold difference between the highest and lowest EC_50_ values. The frequency of the EC_50_ values exhibited a unimodal distribution with a positive skew (Figure 1). The low EC_50_ values also confirmed that no flumorph resistance had yet developed in field populations of *P. sojae* in China.

### 3.2 Selection of flumorph‐resistant *P. sojae* isolates in vitro

The flumorph screening process resulted in the identification of five different types of flumorph‐resistant isolates: Types I, II, III, IV and V. The Type I mutants, which had a low RF (RF = 2) and included isolates RF13‐2‐2 and RF13‐2‐4, were obtained by exposure of the wild‐type isolate Ps13 to a low concentration (2.5 μg/ml) of flumorph (Table 1). No resistant mutants resulted from flumorph adaptation in any of the five other wild‐type isolates tested: Ps4, Ps5, Ps6, Ps8 and Ps10. However, several other resistant types were obtained when the Type I mutants were exposed to higher concentrations, with the Type II, III and IV mutants obtained at 5 μg/ml flumorph, and the Type V mutants, which exhibited the highest level of resistance, from exposure to 100 μg/ml flumorph (Table 1). The selection process was repeated to confirm the results. A similar pattern of mutation was observed, with Type II, III, IV and V mutants obtained. Taken together, these results indicate that the evolution of isolates highly resistant to flumorph could be a two‐step process via the Type I intermediary.

**Table 1:** Profiles of five types of flumorph‐resistant *Phytophthora sojae* mutants and their sensitivity to three other CAAs

Type	Isolate	Flumorph EC_50_ (μg/ml) ^a^					RF ^b^			
		1st	3rd	5th	7th	10th	FLU	DMM	MPD	IPRO
Parent	Ps13	0.4	0.5	0.5	0.7	1.1	–	–	–	–
Type I	RF13−2–2	1.9	2.0	2.5	2.7	2.5	2	9	1	3
	RF13−2–4	1.9	2.0	2.7	2.8	2.6	2	8	1	2
Type II	RF11	15.8	14.0	24.4	17.5	19.3	17	377	6	16
Type III	RF8	78.5	59.6	62.6	58.8	84.6	76	16	>500	>42
Type IV	RF3	>100	>100	>100	>100	>100	>90	180	>500	>42
	RF5	>100	>100	>100	>100	>100	>90	184	>500	>42
	RF9	>100	>100	>100	>100	>100	>90	146	>500	>42
Type V	RF1	>100	>100	>100	>100	>100	>90	>769	>500	>42

Abbreviations: DMM, dimethomorph; FLU, flumorph; IPRO, iprovalicarb; MPD, mandipropamid.

^a^ 1st, 3rd, 5th, 7th and 10th indicate the number of subcultures on flumorph‐free media.

^b^ RF, Resistance factor = EC_50_ of mutants at the 10th transfer /EC_50_ of its parent at the 10th transfer.

### 3.3 Analysis of the *CesA3* gene in *P. sojae*

**Figure 2:**
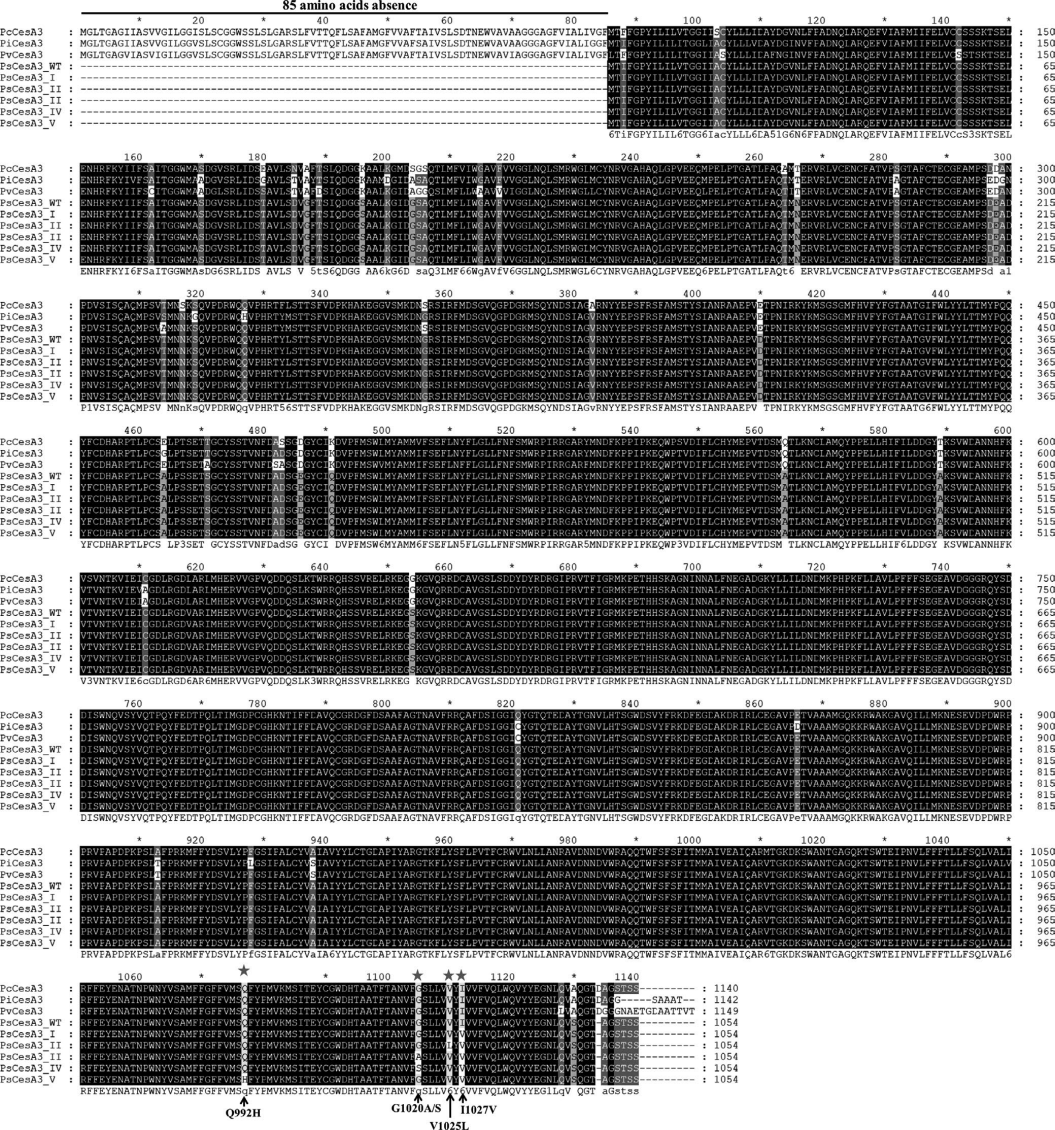
Five types of flumorph‐resistant *Phytophthora sojae* mutants carrying different amino acid substitutions in their CesA3 proteins. PcCesA3, PiCesA3, PvCesA3 and PsCesA3‐WT are the CesA3 amino acids from *Phytophthora capsici* (GenBank: AFB20353.1), *Phytophthora infestans* (GenBank: ABP96904.1), *Plasmopara viticola* (GenBank: ADD84672.1) and *P. sojae* (GenBank: ABP96908.1), respectively. PsCesA3 lacked an 85 amino acid segment from its N‐terminus compared to the sequences from the other oomycete species

The full‐length *CesA3* gene was successfully cloned from both the sensitive wild‐type isolates and the five mutant types. The *CesA3* gene of *P. sojae* was found to be 3165 bp in length and contained no introns. The deduced amino acid sequence of the PsCesA3 protein (GenBank: ABP96908.1) contained 1054 amino acids, which was significantly shorter than the sequences from *P. capsici* (PcCesA3; GenBank: AFB20353.1), *P. infestans* (PiCesA3; GenBank: ABP96904.1) and *P. viticola* (PvCesA3; GenBank: ADD84672.1). Further analysis indicated that the sequence from *P. sojae* lacked an 85–amino acid segment from its N‐terminus compared to the sequences from the other oomycete species (Figure 2).

Multiple sequence alignment of the PsCesA3 protein sequences from the five mutant types and the wild‐type parental isolate revealed that all of the mutants contained an amino acid substitution that resulted in the replacement of an isoleucine residue (ATT) by a valine residue (GTT) at codon 1027 (Figure 2). However, although the Type I mutant carried this single point mutation, the other mutant types were found to contain additional amino acid changes. For example, the Type II mutants contained a substitution at codon 1025, V1025L, while the Type III and IV mutants both contained a second substitution at codon 1020, but involving different amino acids, G1020A and G1020S, respectively (Figure 2). The Type V mutants, which had the highest levels of resistance to flumorph, were also found to carry an addition substitution, in this case at codon 992: Q992H (Figure 2). Considering these results in the context of the sensitivity data for the five mutant types, it becomes apparent that V1025L, G1020A, G1020S and Q992H were able to augment the baseline resistance associated with the I1027V mutation, resulting in increasing levels of resistance in Type II, III, IV and V mutants, respectively.

Comparison with the *CesA3* sequences from other oomycete species, and accounting for the absence of 85 amino acids at the N‐terminus of the *P. sojae* CesA3 protein, revealed that several amino acid changes in the *P. sojae* mutants, including those at positions 1020 and 992, corresponded to equivalent mutations 1105 and 1077, respectively, previously documented in *P. capsici* (Chen et al., 2011; Pang et al., 2013), *P. infestans* (Blum, Boehler, et al., 2010) and *P. viticola* (Blum, Waldner, et al., 2010). As such, only the I1027V (I1112V) and V1025L (V1110L) substitutions constitute newly discovered mutations that have not been associated with CAA resistance in previous studies.

### 3.4 Characterization of flumorph‐resistant isolates

#### 3.4.1 Stability of resistance

The resistant phenotype of the five types was extremely stable, with no differences found between the EC_50_ values of the 3rd, 5th, 7th and 10th‐generation colonies compared to the 1st generation (Table 1).

#### 3.4.2 Resistance to three other CAA fungicides

The five flumorph‐resistant types varied in their sensitivity to other CAA fungicides (Table 1). For example, the Type I mutants, which exhibited a low level of resistance to flumorph, were found to have low resistance to dimethomorph and iprovalicarb, and wild‐type sensitivity to mandipropamid; the Type II mutants, which were moderately resistant to flumorph, exhibited high resistance to dimethomorph, moderately resistance to iprovalicarb, but low resistance to mandipropamid. Similarly, the highly resistant Type III mutants exhibited varying degrees of resistance to other CAA fungicides: high resistance to mandipropamid and iprovalicarb but only moderate resistance to dimethomorph. In contrast, the highly resistant Type IV and V mutants were found to exhibit high levels of resistance to all the CAA fungicides tested, with the Type V mutants being the most resistant.

#### 3.4.3 Mycelial growth, sporulation, cyst germination and virulence in vitro

The five flumorph‐resistant types were also found to vary in their patterns of mycelial growth, sporulation, cytospore germination and virulence compared to the parental isolate Ps13 (Table 2). For example, the Type I, III and IV mutants were found to grow either faster than or at a similar rate to Ps13, while the Type II and V mutants grew significantly slower (*p* < 0.05). Furthermore, the sporulation level of Type II was reduced by nearly two times compared with the parental isolate Ps13, even though the difference was not significant (*p* < 0.05). The cystospore germination of the Type V mutants was also reduced without significant difference. However, there seemed to be no difference between the virulence of any of the mutant types and the parental isolate Ps13, which all produced lesions of a similar length. In summary, these results indicate no fitness cost to the Type I, III and IV mutants, but the other mutants Type II and Type V were negatively affected.

**Table 2:** Fitness parameters of five types of flumorph‐resistant mutants and sensitive isolate Ps13 of *Phytophthora sojae*

Type	Isolate	Colony diameter at 144 h (mm)	Sporulation (×10^5^/cm^2^)	Germination rate	Lesion length on soybean seedlings (mm)
Parent	Ps13	54.0 ± 1.7 cd	4.0 ± 0.6 ab	0.96 ± 0.01 a	51.8 ± 5.7 a
Type I	RF13−2–2	59.4 ± 0.7 ab	5.0 ± 0.6 a	0.95 ± 0.01 a	49.3 ± 3.0 a
	RF13−2–4	58.0 ± 0.5 abc	4.0 ± 0.6 ab	0.97 ± 0.01 a	33.0 ± 4.7 a
Type II	RF11	43.5 ± 1.2 e	2.3 ± 0.3 ab	0.92 ± 0.01 a	37.5 ± 7.1 a
Type III	RF8	60.4 ± 0.4 ab	3.0 ± 0.6 ab	0.96 ± 0.01 a	40.3 ± 3.3 a
Type IV	RF3	62.5 ± 1.1 a	3.0 ± 0.6 ab	0.96 ± 0.00 a	50.7 ± 2.8 a
	RF5	57.1 ± 1.2 bcd	2.7 ± 0.3 ab	0.95 ± 0.01 a	ND
	RF9	52.5 ± 0.6 d	3.0 ± 0.6 ab	0.93 ± 0.02 a	42.3 ± 5.2 a
Type V	RF1	44.6 ± 0.7 e	3.3 ± 0.3 b	0.91 ± 0.01 a	40.0 ± 9.5 a

Note

Values followed by same letters within the same column indicate no significant differences according to Tukey's honestly significant difference test (Tukey's HSD; *p* = 0.05).

Abbreviation: ND, not detected.

### 3.5 Cross‐resistance to non‐CAA fungicides

**Figure 3:**
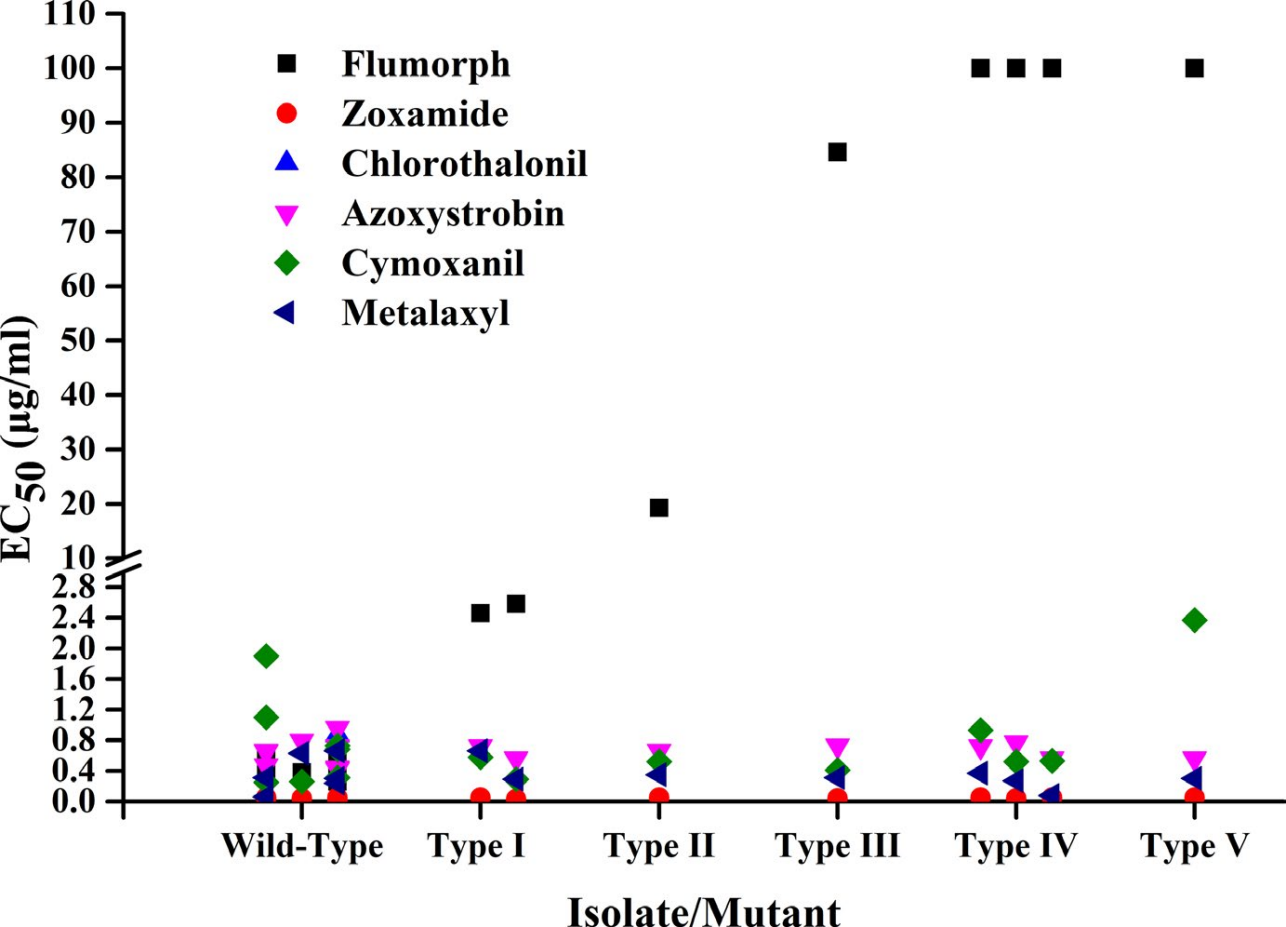
Cross‐resistance between flumorph and five non‐CAA fungicides in five types of flumorph‐resistant mutants

None of the five flumorph‐resistant mutant types were found to exhibit any resistance to other commonly used fungicides—zoxamide (EC_50_ = 0.02–0.05 μg/ml), chlorothalonil (EC_50_ = 0.87–8.56 μg/ml), azoxystrobin (EC_50_ = 0.42–0.96 μg/ml), cymoxanil (EC_50_ = 0.25–5.21 μg/ml), and metalaxyl (EC_50_ = 0.06–0.66 μg/ml)—regardless of their level of resistance to flumorph (Figure 3, Table S4). Given that all of the fungicides tested belonged to groups chemically distinct from CAA fungicides, these results provide strong evidence that there is no cross‐resistance between flumorph and non‐CAA fungicides.

### 3.6 Cas9‐mediated mutagenesis of the *CesA3* gene in *P. sojae*

Site‐directed mutagenesis using CRISPR/Cas9 technology was employed to generate a series of transformants with altered PsCesA3 sequences in the sensitive wild‐type isolate P6497, which was selected because its entire genome has been sequenced. The technique successfully replicated the five mutant types (Types I–V) previously generated by flumorph adaptation, which were designated Types TI, TII, TIII, TIV and TV (Table 3). The Type TI transformants, which were homozygous for I1027V, were found to exhibit a similar resistance profile to the Type I mutants, with low resistance to flumorph, dimethomorph and iprovalicarb, and wild‐type sensitivity to mandipropamid. However, the Type TII transformants (Type TIIa), which were homozygous for both I1027V and V1025L, were found to perform slightly differently than their Type II counterparts, having no resistance to mandipropamid and lower resistance to flumorph and dimethomorph, but similarly moderate resistance to iprovalicarb. The Type TII transformants (Type TIIb) that were heterozygous for I1027V and V1025L exhibited even lower levels of resistance than the homozygous Type TII transformants, which could indicate that CAA resistance in *P. sojae* is a semi‐dominant trait. The homozygous Type TIII (I1027V and G1020A), TIV (I1027V and G1020S) and TV (I1027V and Q992H) transformants all exhibited similar levels of resistance in comparison to their Type III, IV, and V counterparts, with the Type TV transformants again being the most resistant.

**Table 3:** The CAA sensitivities of transformants carrying different amino acid substitutions in their CesA3 proteins

Isolate/Transformant	Number^a^	RF^b^				Amino acid substitutions in CesA3			
		FLU	DMM	MPD	IPRO	Q992H	G1020A/S	V1025L	I1027V
P6497 Parent	1	1	1	1	1	Gln (CAG)	Gly (GGC)	Val (GTG)	Ile (ATT)
Type TI	3	2; 2; 2	4; 4; 4	1; 1; 1	3; 3; 3	Gln (CAG)	Gly (GGC)	Val (GTG)	Val (GTT)
Type TIIa^c^	2	5; 4	6; 7	1; 1	30; 19	Gln (CAG)	Gly (GGC)	Leu (TTG)	Val (GTT)
Type TIIb^c^	1	3	4	1	4	Gln (CAG)	Gly (GGC)	Val/Leu (G/TTG)	Ile /Val (A/GTT)
Type TIII	3	46; 41; 42	38; 38; 34	>1000	>111	Gln (CAG)	Ala (GCC)	Val (GTG)	Val (GTT)
Type TIV	3	>63	212; 194; 206	>1000	>111	Gln (CAG)	Ser (AGC)	Val (GTG)	Val (GTT)
Type TV	3	>63	>300	>1000	>111	His (CAT)	Gly (GGC)	Val (GTG)	Val (GTT)
Type TIIb^‐^ ^c^	2	1; 2	1; 1	1; 1	2; 2	Gln (CAG)	Gly (GGC)	Val/Leu (G/TTG)	Ile (ATT)
Type TIII^−^	3	6; 6; 7	7; 6; 7	>1000	>111	Gln (CAG)	Ala (GCC)	Val (GTG)	Ile (ATT)
Type TIV^−^	3	24; 23; 32	26; 25; 32	>1000	>111	Gln (CAG)	Ser (AGC)	Val (GTG)	Ile (ATT)
Type TV^−^	3	>63	>300	>1000	>111	His (CAT)	Gly (GGC)	Val (GTG)	Ile (ATT)

Abbreviations: DMM, dimethomorph; FLU, flumorph; IPRO, iprovalicarb; MPD, mandipropamid.

^a^ Number of transformants recovered for each mutation Type.

^b^ RF, Resistance factor = EC_50_ of mutants /EC_50_ of parent.

^c^ Type TIIa was a homozygous transformant, while Type TIIb and TIIb^−^ were heterozygous.

Most of the transformants bearing single copies of the V1025L, G1020A, G1020S and Q992H mutations in the absence of I1027V, which were designated Type TII^−^, TIII^−^, TIV^−^ and TV^−^, also exhibited significant levels of resistance to CAA fungicides, although generally to a lower degree than their Type TII, TIII, TIV and TV counterparts (Table 3). The heterozygous Type TII^−^ isolates (Type TIIb^−^) performed poorest, exhibiting wild‐type sensitivity to dimethomorph and mandipropamid, and only low levels of resistance to flumorph and iprovalicarb; no homozygous Type TII^−^ was obtained. The Type TIII^−^ and TIV^−^ transformants exhibited similar levels of resistance to mandipropamid and iprovalicarb but reduced resistance to flumorph and dimethomorph. In contrast, the Type TV^−^ transformants exhibited equivalents levels of resistance, being highly resistant to all the CAA fungicides tested.

In summary, these results indicate that the I1027V mutation in the CesA3 protein of *P. sojae* can confer low‐level resistance to most CAA fungicides, but that higher levels of resistance can be achieved by co‐presence with the V1025L, G1020A/S and Q992H mutations. In addition, the results indicate that the G1020A/S and Q992H mutations in isolation can confer moderate to high levels of resistance to CAA fungicides, although not as effectively as when linked with I1027V, and that the Q992H mutation can confer extremely high levels of resistance to all the CAA fungicides tested, regardless of whether it is linked to the I1027V mutation. These results provide strong evidence that the evolution of isolates highly resistant to CAA fungicides can occur in a two‐step process via the Type I (I1027V) intermediary.

### 3.7 Biological characteristics of CRISPR/Cas9 transformants

Most of the transformants produced by site‐directed mutation were found to have significantly impaired levels (*p* < 0.05) of mycelial growth, sporulation, cystospore germination and virulence compared to the P6497 parental isolate, with the Type TV transformants again the most affected (Table 4). However, the biological characteristics of the transformants were actually comparable or significantly better (*p* < 0.05) than those of another wild‐type isolate, R6. These results indicate that although flumorph resistance incurs a fitness penalty in *P. sojae*, resistant isolates can still remain competitive with other field isolates.

**Table 4:** Biological characteristics of two wild‐type isolates and ten transformants carrying different amino acid substitutions in their CesA3 proteins

Isolate/Transformant	Colony diameter at 144 h (mm)	Sporulation (×10^4^/cm^2^)	Germination rate	Lesion length on seedlings (cm)
P6497 Parent	50.5 ± 0.2 abc	28.8 ± 1.7 a	0.85 ± 0.03 a	4.0 ± 0.7 a
R6	35.8 ± 0.1 f	1.3 ± 0.3 f	0.50 ± 0.07 cde	2.9 ± 0.2 ab
Type TI	50.0 ± 0.2 abc	14.8 ± 2.6 cde	0.61 ± 0.03 bcd	1.2 ± 0.2 b
Type TIIa	40.1 ± 1.3 f	10.9 ± 1.1 cdef	0.55 ± 0.04 bcde	0.9 ± 0.08 b
Type TIIb	45.4 ± 0.5 d	18.5 ± 3.7 bcd	0.55 ± 0.05 bcde	0.9 ± 0.2 b
Type TIII	49.7 ± 0.1 bc	10.8 ± 0.9 def	0.45 ± 0.04 de	2.3 ± 0.6 ab
Type TIV	50.8 ± 0.5 ab	20.9 ± 1.7 abc	0.74 ± 0.02 ab	1.6 ± 0.5 ab
Type TV	43.2 ± 0.3 e	6.7 ± 1.1 ef	0.38 ± 0.05 e	2.1 ± 0.5 ab
Type TIIb^−^	46.7 ± 0.6 d	6.5 ± 1.0 ef	0.61 ± 0.04 bcd	1.1 ± 0.1 b
Type TIII^−^	48.5 ± 0.3 c	20.3 ± 1.5 abcd	0.71 ± 0.03 ab	2.0 ± 0.5 ab
Type TIV^−^	51.9 ± 0.7 a	13.8 ± 2.2 cde	0.66 ± 0.02 abc	2.5 ± 0.4 ab
Type TV^−^	44.2 ± 0.3 de	25.1 ± 2.1 ab	0.69 ± 0.03 abc	1.7 ± 0.4 ab

Note

Type TIIa was a homozygous transformant, while Type TIIb and TIIb^−^ were heterozygous. Values followed by same letters within the same column indicate no significant differences according to Tukey's honestly significant difference test (Tukey's HSD; *p* = 0.05).

## 4 DISCUSSION

The current study evaluated the resistance development process of *P. sojae* to the CAA fungicide flumorph, and the genetics associated with the resistance mechanism. Since no resistant field isolate was detected, six wild‐type *P. sojae* isolates were randomly selected for screening experiments to generate flumorph‐resistant mutants. However, only one (Ps13) of the six strains evolved resistance during the selection process. The mechanism by which the wild‐type strains behave differently is complicated. Similar observations have also been reported in the selection process of *P. capsici* resistance to oxathiapiprolin (2 out of 12 wild‐type strains, each of which has evolved a mutation type; Miao et al., 2016), *P. sojae* resistance to zoxamide (1 out of 10 wild‐type strains; Cai et al., 2016), *Botrytis cinerea* resistance to pyrisoxazole (one out of four wild‐type strains; Zhang, Imran, et al., 2020) and zoxamide (one out of eight wild‐type strains; Cai et al., 2015), and *Magnaporthe oryzae* resistance to a novel pyrimidine amine compound SYP‐34773 (1 out of 10 wild‐type strains; Zhang, Meng, et al., 2020). The mutation rate is variable between different organisms and between different individuals of the same species. It is reported that physiological conditions are often implicated to contribute to mutation rate variation in organisms (Baer, 2008; Saxena et al., 2019). For example, the concentration of free radicals, the induction of error‐prone DNA‐polymerases mediated by SOS response and the imbalanced nucleotide metabolism are reported to correlates with mutation rate (Rodríguez‐Rojas et al., 2013). It is worth to illuminate the potential mechanisms why the strains behave differently under the same selection pressure, as an area for future research. In addition, our further investigation in the fitness properties showed that the resistant isolates evolved from Ps13 remain survival ability and can be competitive with field isolates. It means that they can evolve into a dominant population under flumorph selection pressure.

**Figure 4:**
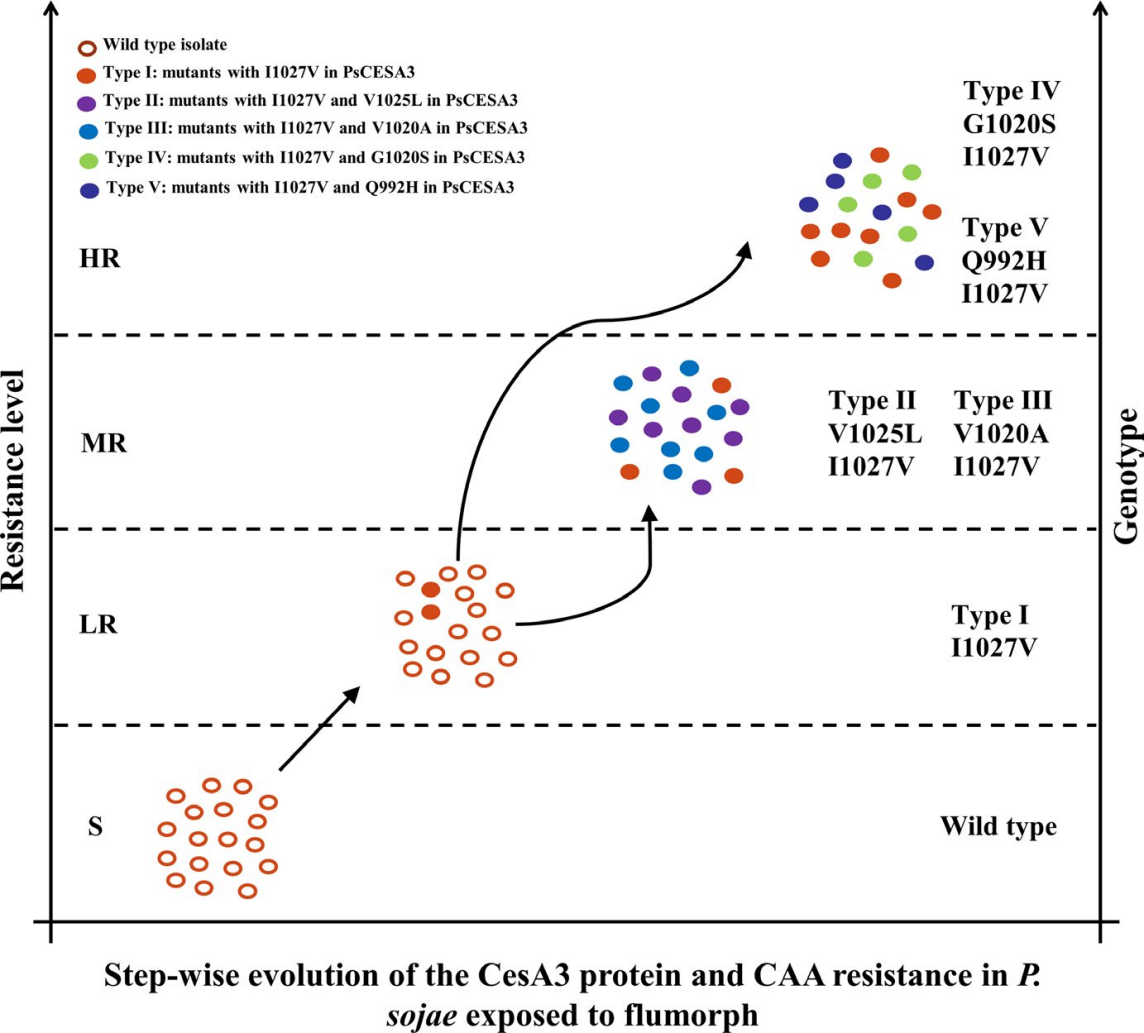
Stepwise evolution of the CesA3 protein and CAA resistance in *Phytophthora sojae* exposed to flumorph. The left vertical axis indicates the resistance level to flumorph, while the right vertical axis indicates the genotypes of six types of isolates. “S” indicates sensitive to flumorph, “LR” low resistance, “MR” moderate resistance, “HR” high resistance. The arrows indicate the application of low doses of flumorph could result in the selection of the low resistance Type I mutants, but that raising the dosage to maintain comparable levels of control could elicit the rapid evolution of the more resistant Type II, III, IV and V mutants

A total of five mutant types with varying levels of resistance was produced via adaptation on a flumorph‐amended medium. Although no mutants were obtained by direct exposure to high concentrations of flumorph (5–100 μg/ml), Type I mutants, which exhibited a low level of resistance, were obtained at a low concentration (2.5 μg/ml). The other mutants (Types II, III, IV and V), which exhibited higher levels of resistance to CAA fungicides, were produced by subjecting the Type I mutants to higher concentrations of flumorph. Similar results were obtained when the entire screening process was repeated, which indicates that the evolution of resistance to flumorph in *P. sojae* could be a two‐step process, where high resistance phenotypes could develop indirectly from low resistance ones (Figure 4). These results indicate that the application of low rates of flumorph in the field could result in the selection of the low resistance Type I mutants, but that raising the dosage to maintain comparable levels of control could elicit the rapid evolution of the more resistant Type II, III, IV and V mutants, which would render flumorph ineffective as a method of control. However, the observation that high concentrations of flumorph did not result in the selection of any resistant mutants could indicate that initial applications of flumorph at high rates could in fact reduce the risk of resistance developing in field populations. Unfortunately, such a strategy could be difficult to apply in practice because under natural conditions plant pathogens are frequently exposed to low concentrations of pesticides, either as a consequence of an aquatic or soil environment, or as a result of pesticide degradation. Similar observations have also been made during medical research, with many studies reporting that exposure to low concentrations of antibiotics is an important factor leading to the enrichment and maintenance of antibiotic resistance within bacterial populations (Andersson & Hughes, 2014; Gullberg et al., 2011; Liu et al., 2011; Toprak et al., 2012; Wistrand‐Yuen et al., 2018). For example, the resistance of *Escherichia coli* to the antibiotics trimethoprim from low to high level was reported to evolve in a stepwise manner with sequential accumulations of mutations in the target protein (Toprak et al., 2012). Therefore, the phenomenon described in the current study could have broader biological significance.

Previous studies have shown that the cellulose synthase encoded by the *CesA3* gene is the target protein for CAA fungicides, and that resistance can be conferred by single amino acid substitutions (Blum & Gisi, 2012). For example, several studies have shown that a multitude of changes to the conserved glycine residue at codon 1105 of the CesA3 protein can result in CAA resistance in oomycete pathogens (Blum, Boehler, et al., 2010; Blum, Waldner, et al., 2010; Chen et al., 2011). The results of the current study also provided evidence that a wide range of mutations in the CesA3 protein could be linked to CAA resistance in *P. sojae*. Perhaps of greatest interest was the I1027V mutation that was found in all of the mutants tested, but that was particularly characteristic of the Type I mutants, which only exhibited low resistance to CAA fungicides. The four other mutant types were found to contain secondary mutations: V1025L in Type II, G1020A in Type III, G1020S in Type IV and Q992H in Type V. Sensitivity assays indicated that the presence of these additional mutations significantly increased resistance to CAA fungicides, with the V1025L mutation providing low levels of resistance, G1020A and G1020S moderate to high levels, and Q992H the highest resistance of all. However, when the shorter CesA3 sequence of *P. sojae*, which lacked an 85 segment at its N‐terminus, was compared with the sequences from *P. infestans*, *P. viticola* and *P. cubensis*, it became apparent that two mutations at codons 1020 and 992 corresponded to mutations already documented in the other species, at positions 1105 and 1077, respectively, and that two, I1027V (I1112V) and V1025L (V1110L), constituted newly discovered mutations that had not been documented in other oomycete species. Among these mutations, G1020A/S (G1105A/S) has been found in field isolates (Blum, Waldner, et al., 2010; Chen et al., 2011). Laboratory adaptation experiment provides a more rapid and effective way to study the development and mechanism of fungicide resistance, although it is different from selection under field conditions. Furthermore, many cases have proved that resistance is likely to develop in field and involve the same mechanisms as that selected in laboratory. For example, the resistance to quinone outside inhibitor (Qoi) fungicides caused by G143A mutation in *cyt b* gene is common in field and also often reported in laboratory (Gisi & Sierotzki, 2015). Laboratory resistance is useful for estimating field resistance and plays an important role in establishment of a resistance management strategy.

Site‐directed mutagenesis using CRISPR/Cas9 technology validated the role of the *P. sojae* mutations in CAA resistance, demonstrating that the presence of the I1027V mutation in the resulting transformants conferred low levels of resistance to flumorph, which could be increased when augmented by the secondary V1025L, G1020A, G1020S and Q992H mutations. These results provide further evidence that the evolution of flumorph resistance in *P. sojae* could be a stepwise process. In addition, the current study also demonstrated that the V1025L, G1020A, G1020S and Q992H mutations could also confer resistance to CAA fungicides in the absence of the I1027V mutation. However, with the exception of Q992H, the level of resistance to flumorph and dimethomorph in these transformants was less than when the V1025L and G1020A/S mutations occurred in combination with I1027V. Interestingly, though, their level of resistance to mandipropamid and iprovalicarb was equivalent to that obtained in combination with I1027V. It is reported that inherent activity of each CAA chemical group can vary considerably (Gisi & Sierotzki, 2015). These discrepancies likely reflect the fact that the original mutants were screened via adaptation to flumorph. However, the observation that the Q992H mutation could cause high levels of resistance to all the CAA fungicides tested, even in the absence of I1027V, could indicate that it is a more critical component of the CAA target site in the CesA3 of *P. sojae*, which could overshadow any impact of the I1027V mutation.

Although the biological characteristics of the original mutants differed little compared to their parental isolate Ps13, it is interesting to note that almost all of the transformants exhibited reduced fitness compared to their parental isolate, P6497. However, despite their reduced performance, the transformants also exhibited comparable or greater fitness compared to the wild‐type isolate R6.

Taken together, the results indicate that the resistance evolution of flumorph in *P. sojae* could be a stepwise process, where high resistance isolates could develop indirectly from low resistance ones at a high mutation frequency. And, although flumorph resistance incurs a fitness penalty in *P. sojae*, resistant isolates can still remain competitive with other field isolates. The resistance risk of *P. sojae* to flumorph is suggested to be low to moderate. A precautionary resistance management strategy should be implemented.

## CONFLICTS OF INTEREST

All the authors declare there is no conflict of interest.

## Supporting information

Supplementary Material

## Data Availability

All data supporting the findings of this study are available within the article and the Supplementary material.
