#  Corrigendum

**DOI:** 10.1111/eva.13458

**Published:** 2022-08-15

In Evolutionary Applications 14:4, the article by Cai et al. (2021) contains an error in the analysis of the full length of *CesA3* gene in *Phytophthora sojae*. A segment was previously reported missing at the N‐terminus of *CesA3* gene. The *CesA3* gene of *P. sojae* was found to be 3563 bp in full length, rather than 3165 bp, and contained an intron of 143 bp. The deduced sequence of the PsCesA3 protein contained 1139 amino acids. The mutation sites were thus presented incorrectly. Q992H, G1020A/S, V1025L, and I1027V should be corrected to Q1077H, G1105A/S, V1110L, and I1112V, respectively. The correct Figures 2 and 4 are as follows:

**FIGURE 2:**
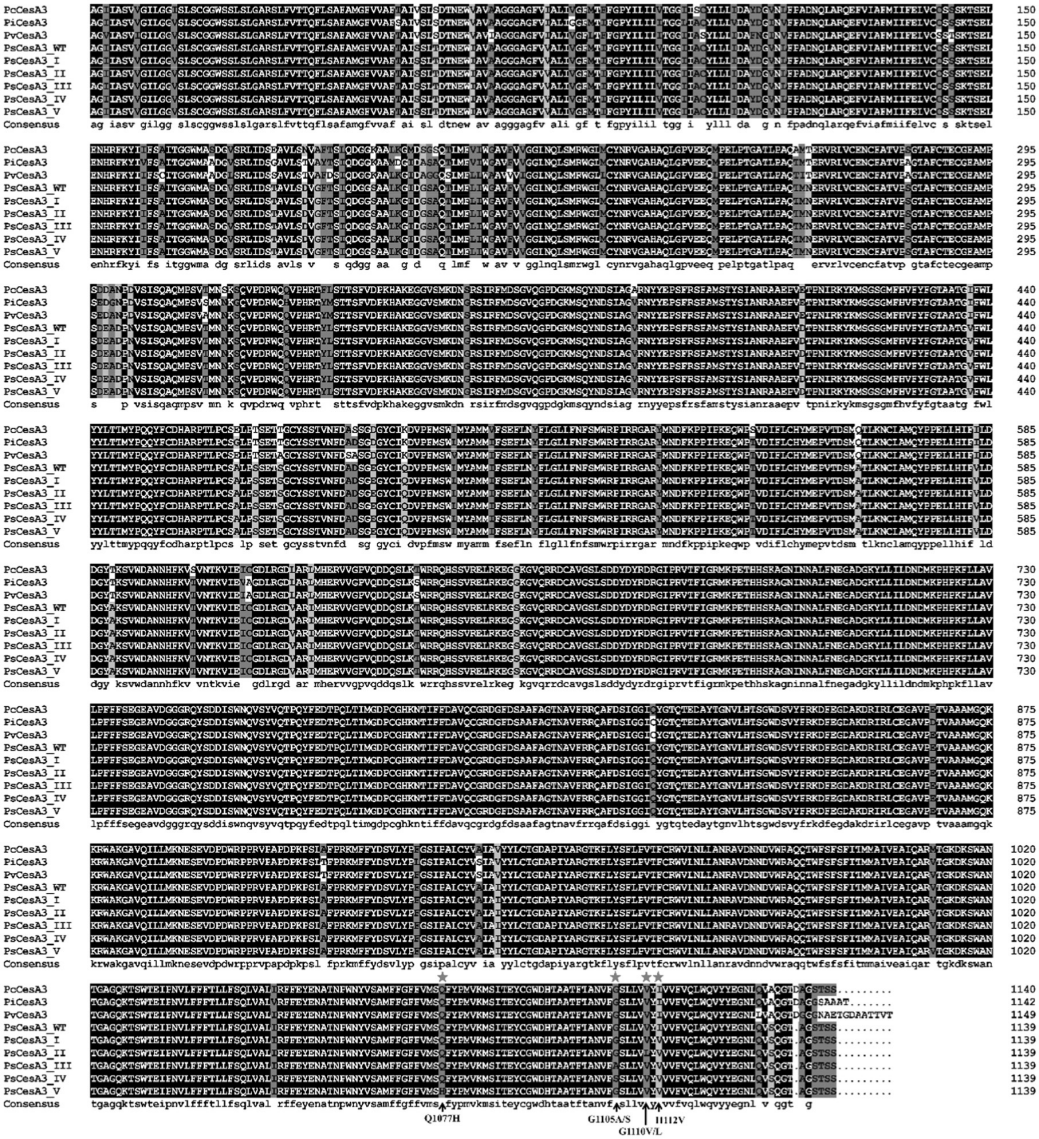
Five types of flumorph‐resistant *Phytophthora sojae* mutants carrying different amino acid substitutions in their CesA3 proteins. PcCesA3, PiCesA3, PvCesA3, and PsCesA3‐WT are the CesA3 amino acids from *Phytophthora capsici* (GenBank: AFB20353.1), *Phytophthora infestans* (GenBank: ABP96904.1), *Plasmopara viticola* (GenBank: ADD84672.1), and *P. sojae* (GenBank: XP_009534128.1), respectively

**FIGURE 4:**
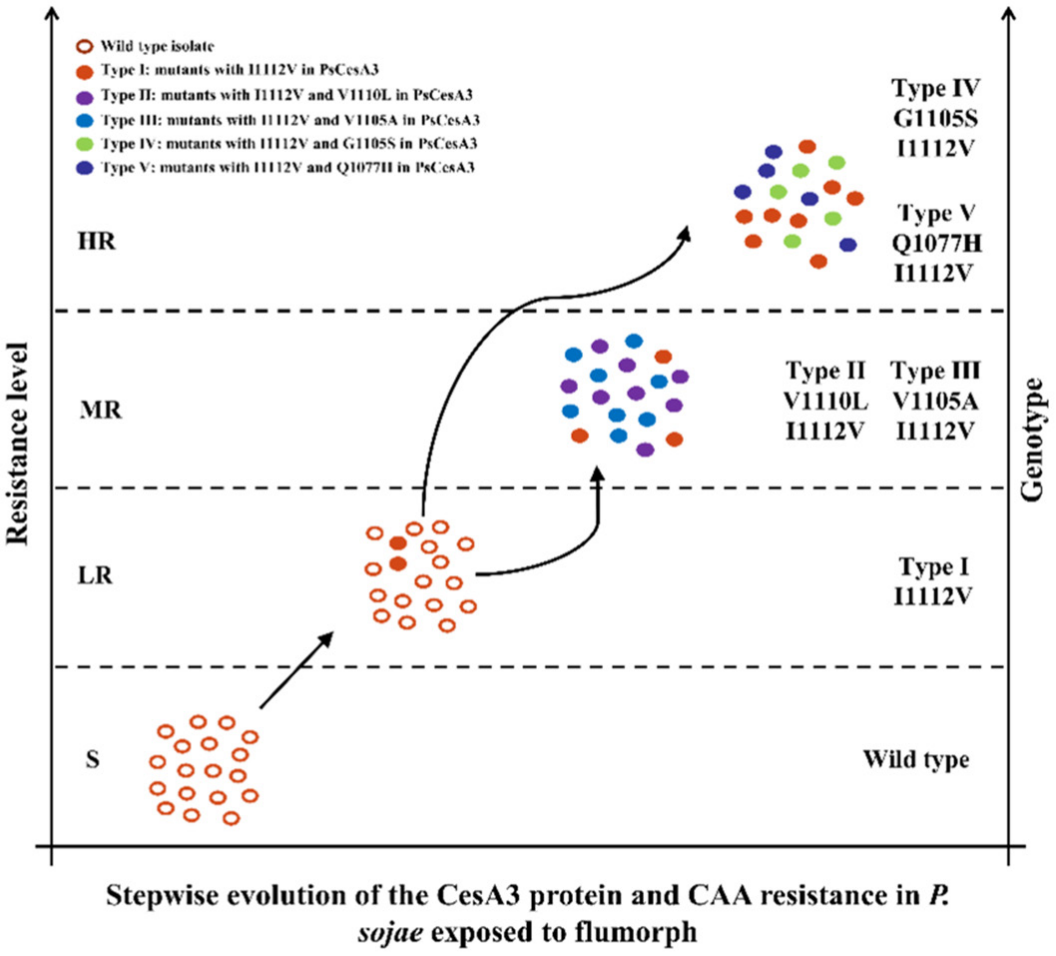
Stepwise evolution of the CesA3 protein and CAA resistance in *P. sojae* exposed to flumorph. The left vertical axis indicates the resistance level to flumorph, while the right vertical axis indicates the genotypes of six types of isolates. “S” indicates sensitivity to flumorph, “LR” low resistance, “MR” moderate resistance, and “HR” high resistance. The arrows indicate the application of low doses of flumorph could result in the selection of the low‐resistance type I mutants, but that raising the dosage to maintain comparable levels of control could elicit the rapid evolution of the more resistant type II, III, IV, and V mutants

The online version of this article has been corrected accordingly.

The authors would like to apologize for this error and for any confusion it may have caused.
