# Supplementary material for: Stepwise accumulation of mutations in CesA3 in Phytophthora sojae results in increasing resistance to CAA fungicides
Source: Evol Appl. 2020 Dec 31;14(4):996–1008. doi: 10.1111/eva.13176 (PMC8061276; doi:10.1111/eva.13176)
Supplement: Supplementary file 1 — Supplementary Material [file EVA-14-996-s001.docx]

**Table S1 The origin of *P. sojae* isolates collected from various provinces of China, and their sensitivities to flumorph.**

| **Code** | **Location** | **Number of strains** | **Coordinates** | **EC_50_ range (μg/ml)** | **Year** |
| --- | --- | --- | --- | --- | --- |
| **Ps1-8** | **Fujian** | **7** | **24.78, 118.08** | **0.30-0.79** | **2008** |
| **Ps9-19** | **Fujian** | **5** | **24.37, 117.91** | **0.24-0.65** | **2002** |
| **Ps41-1, PsJMS2, PsJMSD-2** | **Heilongjiang** | **3** | **46.80, 130.32** | **0.43-0.71** | **1996,2010** |
| **LS-** | **Heilongjiang** | **20** | **45.12, 130.70** | **0.32-0.87** | **2010** |
| **HLYF-** | **Heilongjiang** | **9** | **46.89, 126.08** | **0.22-0.75** | **2010** |
| **SH-** | **Heilongjiang** | **6** | **46.65, 126.98** | **0.56-0.87** | **2010** |
| **CX-** | **Heilongjiang** | **15** | **46.03, 126.61** | **0.18-0.81** | **2010** |
| **DZT1-** | **Heilongjiang** | **10** | **44.57, 129.63** | **0.33-0.65** | **2010** |
| **DZT2-** | **Heilongjiang** | **10** | **44.57, 129.63** | **0.45-0.99** | **2010** |
| **DZT3-** | **Heilongjiang** | **11** | **44.57, 129.63** | **0.30-0.75** | **2004, 2010** |
| **AH-** | **Anhui** | **16** | **32.36, 117.38** | **0.32-0.56** | **2010** |
| **Ps53, FJEL, PsFJ, PsFJ3, PsJN4, PsMG, PsN15, PsN3** | **Fujian; Courtesy of Prof. Zhengdong Zhu, Chinese Academy of Agricultural Sciences** | **8** | **--** | **0.21-0.64** | **--** |
| **Total** |  | **120** |  | **0.18-0.99** | **1996-2010** |

**Table S2 Fungicide concentrations used to determine the sensitivities of wild-type *P. sojae* isolates and five Types of flumorph-resistant mutants to different fungicides.**

| **Fungicide** | **Concentrations (μg/ml)** | |
| --- | --- | --- |
|  | **Wild-type isolates** | **Flumorph-resistant mutants** |
| **Flumorph** | **0, 0.2, 0.3, 0.4, 0.5, 06, 0.8** | **0, 1, 2, 4, 8, 12, 16, 20, 25 or 0, 0.4, 0.6, 0.8, 1, 2, 4** |
| **Dimethomorph** | **0, 0.08, 0.1, 0.12, 0.14, 0.16, 0.18, 0.2** | **0, 2.5, 5, 10, 20, 33 or 0, 0.4, 0.6, 0.8, 1, 2** |
| **Mandipropamid** | **0, 0.008, 0.01, 0.012, 0.014, 0.016, 0.018, 0.02** | **0, 0.008, 0.01, 0.012, 0.014, 0.016, 0.018, 0.02, 0.03, 0.04, 10** |
| **Iprovalicarb** | **0, 0.01, 0.02, 0.04, 0.08, 0.1, 0.12, 0.14, 0.16, 0.18** | **0, 0.2, 0.3, 0.4, 0.5, 0.6, 0.7, 0.8, 1, 10** |
| **Zoxamide** | **0, 0.02, 0.03, 0.04, 0.05, 0.06, 0.07, 0.1** | **0, 0.02, 0.03, 0.04, 0.05, 0.06, 0.07, 0.1** |
| **Metalaxyl** | **0, 0.15, 0.3, 0.6, 1.2, 2.4** | **0, 0.15, 0.3, 0.6, 1.2, 2.4** |
| **Azoxystrobin** | **0, 0.2, 0.4, 0.8, 1.0, 1.2** | **0, 0.2, 0.4, 0.8, 1.0, 1.2** |
| **Cymoxanil** | **0, 0.1, 0.3, 0.5, 1.0, 3.0, 5.0** | **0, 0.1, 0.3, 0.5, 1.0, 3.0, 5.0** |
| **Chlorothalonil** | **0, 2.0, 4.0, 6.0, 8.0, 10.0** | **0, 2.0, 4.0, 6.0, 8.0, 10.0** |

**Table S3 Primers used in the current study.**

| **Primer name** | **Sequence 5'-3'** | **Purpose** |
| --- | --- | --- |
| **PsCesa3PF** | **ATGACGATCTTCGGACCG** | **Amplification of the *CesA3* gene from *P. sojae*. The annealing temperature is 58 ^o^C.** |
| **PsCesA3PR** | **CTAAGACGAAGTGGAACCT** |  |
| **PsCesA3.598F** | **GCCTTCTGTACCGAGTGTGG** | **Sequencing primer for the *P. sojae CesA3* gene.** |
| **PsCesA3.1249F** | **GCGATGATGATCTTCAGTGAG** |  |
| **PsCesA3.1896F** | **TAACGATATGAAGCCGCACC** |  |
| **PsCesA3.2546F** | **TGTGTTACGTGGCTATCGCT** |  |
| **sgRNA4.F** | **CTAGCTATCACCTGATGAGTCCGTGAGGACGAAACGAGTAAGCTCGTCGTGATACTCATCTTGACCAT** | **Oligos for annealing PCR for sgRNA4** |
| **sgRNA4.R** | **AAACATGGTCAAGATGAGTATCACGACGAGCTTACTCGTTTCGTCCTCACGGACTCATCAGGTGATAG** |  |
| **sgRNA8.F** | **CTAGCTTCGTCCTGATGAGTCCGTGAGGACGAAACGAGTAAGCTCGTCGACGAAGAAGCCGAAGAACA** | **Oligos for annealing PCR for sgRNA8** |
| **sgRNA8.R** | **AAACTGTTCTTCGGCTTCTTCGTCGACGAGCTTACTCGTTTCGTCCTCACGGACTCATCAGGACGAAG** |  |
| **sgRNA3.F** | **CTAGCTCTCGTCTGATGAGTCCGTGAGGACGAAACGAGTAAGCTCGTCACGAGAACGCCACGAACCCG** | **Primers for amplifying the sgRNA3.** |
| **sgRNA3.R** | **AAACCGGGTTCGTGGCGTTCTCGTGACGAGCTTACTCGTTTCGTCCTCACGGACTCATCAGACGAGAG** |  |
| **PsCesA3.HDT.F1** | **AGCGAGGTGGACCCAGACT** | **Primers for amplifying the homologous donor template.** |
| **PsCesA3.HDT.R1** | **CAAGTTACGAGCTGGTTAGTGC** |  |
| **sgRNA4.HDT.MF** | **CCAGCCACAGTACTCCGTGATCGACATTTTCACCATCGGGTAGAACTGGCTCATGACG** | **Primers for mediating non-sense mutations in the sgRNA region of each pBSII-PsCesA3.HDT plasmid.** |
| **sgRNA8. HDT.MF** | **CCATAGGGTAGAACTGGCTCATCACAAAAAATCCAAAAAACATCGCAGACACGTAGTTCCACGGGT** |  |
| **sgRNA3. HDT.MF** | **GGCAGACACGTAGTTCCAGGGATTGGTCGCATTTTCATACTCGAAGAATCGGATCAGC** |  |
| **RPL41_Pseq_F** | **CAAGCCTCACTTTCTGCTGACTG** | **Sequencing primer for pYF2.3G-Ribo-sgRNA.** |
| **PsCesA3.A3079G.F** | **TGCACGAATACCACAACGTACACCACCAGCAGC** | **Primers for mediating the A3079G mutation in the pBSII-PsCesA3.HDT plasmid.** |
| **PsCesA3.A3079G.R** | **GCTGCTGGTGGTGTACGTTGTGGTATTCGTGCA** |  |
| **PsCesA3.G2976C.F** | **TTGACCATAGGGTAGAAGTGGCTCATGACGAAGAA** | **Primers for mediating the G2976C mutation in the pBSII-PsCesA3.HDT plasmid.** |
| **PsCesA3.G2976C.R** | **TTCTTCGTCATGAGCCACTTCTACCCTATGGTCAA** |  |
| **PsCesA3.G3059C.F** | **CACCAGCAGCGAGGCGAACACGTTGGC** | **Primers for mediating the G3059C mutation in the pBSII-PsCesA3.HDT plasmid.** |
| **PsCesA3.G3059C.R** | **GCCAACGTGTTCGCCTCGCTGCTGGTG** |  |
| **PsCesA3.G3058A.F** | **GGCCAACGTGTTCAGCTCGCTGCTGGT** | **Primers for mediating the G3058A in the pBSII-PsCesA3.HDT plasmid.** |
| **PsCesA3.G3058A.R** | **ACCAGCAGCGAGCTGAACACGTTGGCC** |  |
| **PsCesA3.G3073T.F** | **AATACCACAATGTACAACACCAGCAGCGAGCCG** | **Primers for mediating the G3073T mutation in the pBSII-PsCesA3.HDT plasmid.** |
| **PsCesA3.G3073T.R** | **CGGCTCGCTGCTGGTGTTGTACATTGTGGTATT** |  |

Table S4 Cross resistance between flumorph and five non-CAA fungicides in five Types of flumorph-resistant mutant and eight wild-type isolates.

| **Isolate/Mutant** | | **EC_50_ (μg/ml)** | | | | | |
| --- | --- | --- | --- | --- | --- | --- | --- |
|  |  | **Flumorph** | **Zoxamide** | **Chlorothalonil** | **Azoxystrobin** | **Cymoxanil** | **Metalaxyl** |
| **Wild-type** | **Ps13** | **0.38** | **0.04** | **7.85** | **0.79** | **0.26** | **0.63** |
|  | **JMS2** | **0.55** | **0.05** | **3.74** | **0.47** | **0.25** | **0.06** |
|  | **Ps6** | **0.69** | **0.04** | **6.54** | **0.44** | **0.68** | **0.30** |
|  | **PsFJ3** | **0.37** | **0.04** | **5.89** | **0.42** | **5.21** | **0.24** |
|  | **PsJSJ-07-2** | **0.31** | **0.04** | **5.77** | **0.65** | **1.10** | **0.06** |
|  | **PsJN4** | **0.53** | **0.05** | **5.64** | **0.72** | **0.31** | **0.66** |
|  | **Ps4** | **0.26** | **0.02** | **0.87** | **0.96** | **0.73** | **0.30** |
|  | **AH1204** | **0.45** | **0.04** | **7.00** | **0.66** | **1.90** | **0.31** |
| **Type I** | **RF13-2-2** | **2.46** | **0.05** | **5.64** | **0.72** | **0.58** | **0.66** |
|  | **RF13-2-4** | **2.58** | **0.03** | **4.89** | **0.56** | **0.29** | **0.29** |
| **Type II** | **RF11** | **19.27** | **0.05** | **3.05** | **0.66** | **0.52** | **0.35** |
| **Type III** | **RF8** | **84.61** | **0.04** | **5.62** | **0.73** | **0.41** | **0.31** |
| **Type IV** | **RF3** | **>100** | **0.04** | **3.39** | **0.77** | **0.52** | **0.27** |
|  | **RF5** | **>100** | **0.05** | **6.50** | **0.56** | **0.53** | **0.08** |
|  | **RF9** | **>100** | **0.05** | **4.89** | **0.72** | **0.93** | **0.37** |
| **Type V** | **RF1** | **>100** | **0.05** | **8.56** | **0.56** | **2.37** | **0.30** |


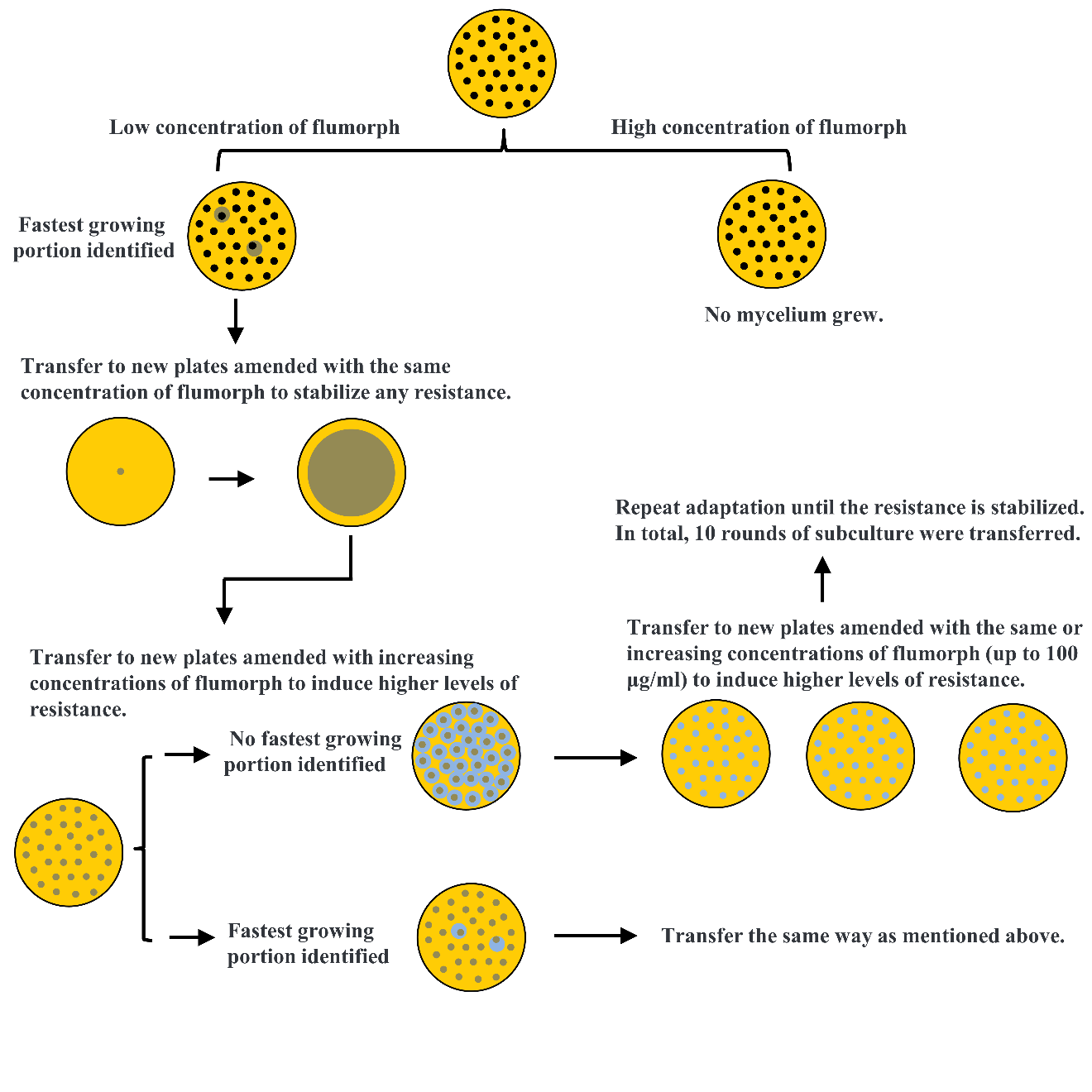


**Figure S1 The schematic of the mutant screening experiments**


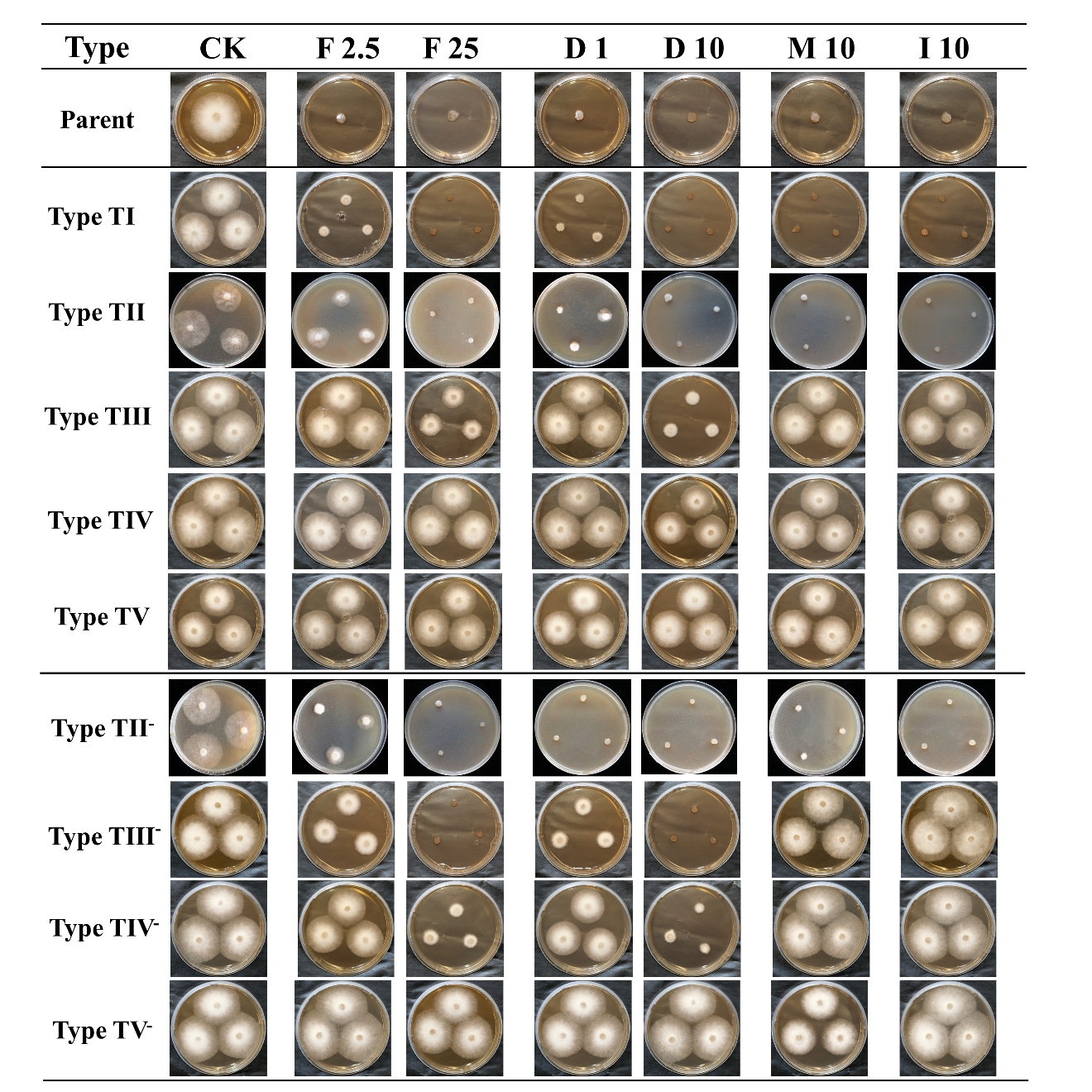


**Figure S2 Mycelia growth of the nine types of *P. sojae* transformants on CAAs-amended medium at various concentrations. F2.5: 2.5 μg/ml flumorph; F25: 25 μg/ml flumorph; D1: 1 μg/ml dimethomorph; D10: 10 μg/ml dimethomorph; M10: 10 μg/ml mandipropamid; I10: 10 μg/ml iprovalicarb.**


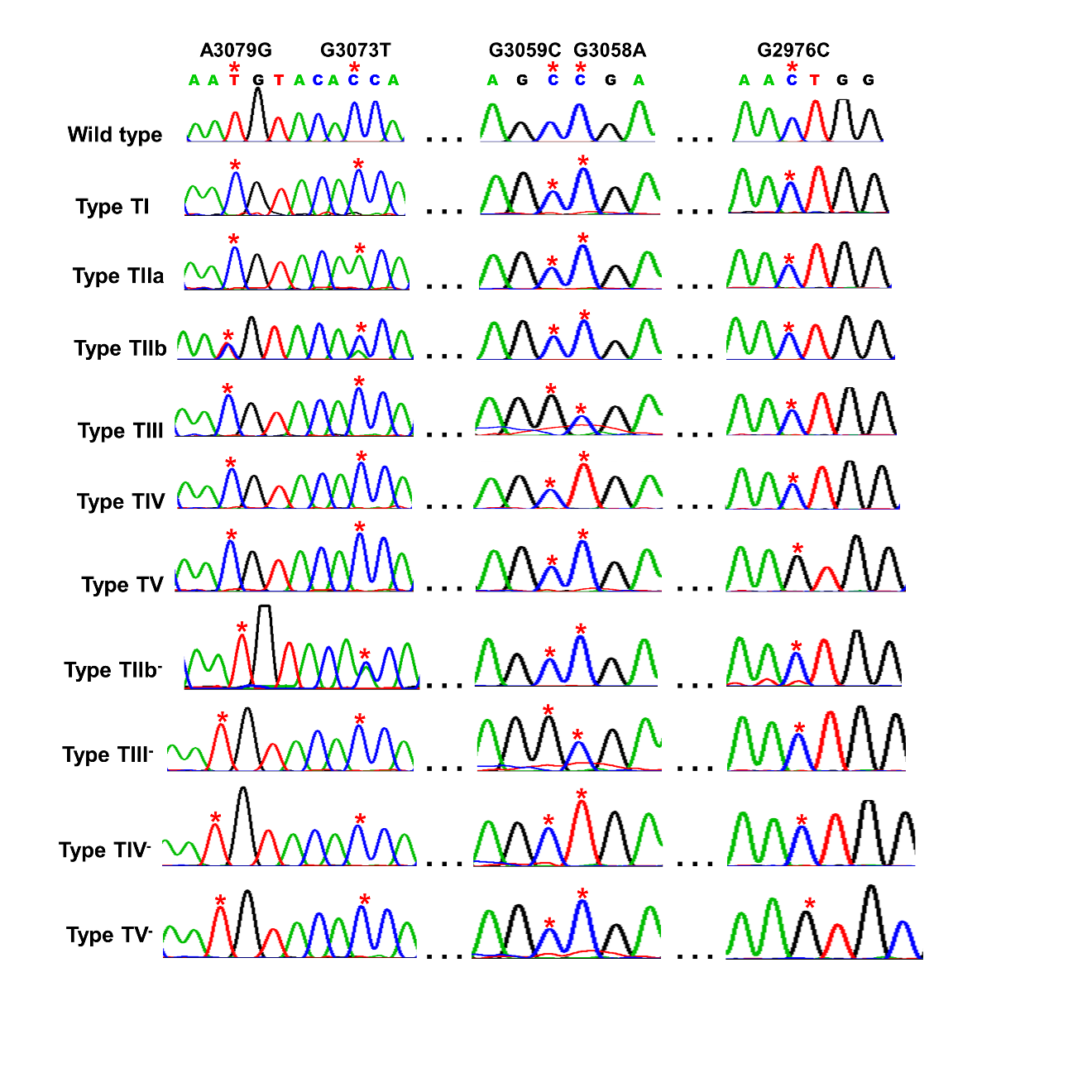


**Figure S3 Sequencing chromatograms of the ten types of mutations in the single zoospore-purified transformants and the wild type parental isolate. The chromas sequence is reverse complementary, and the red asterisk (*) indicates the bases complementary to the mutated bases A3079G, G3073T, G3059C, G3058A or G2976C listed in the first line. The sequencing profiles indicate that these transformant lines are all homozygous except Type TIIb and Type TIIb^-^ which are heterozygous.**
